# Factors affecting the effectiveness of the physical activity counselling intervention implemented in primary health care in adults with type 2 diabetes

**DOI:** 10.1186/s12902-023-01428-w

**Published:** 2023-08-07

**Authors:** Tuula Martiskainen, Marja-Leena Lamidi, Mika Venojärvi, Heikki Tikkanen, Tiina Laatikainen

**Affiliations:** 1 Joint municipal authority for North Karelia social and health services (Siun sote), Tikkamäentie 16, Joensuu, 70210 Finland; 2 Institute of Public Health and Clinical Nutrition, University of Eastern Finland, PO BOX 1627, Kuopio, 70211 Finland; 3 Institute of Biomedicine/Sports and Exercise Medicine, University of Eastern Finland, PO BOX 1627, Kuopio, 70211 Finland; 4 Department of Public Health and Social Welfare, Finnish Institute for Health and Welfare, PO BOX 30, Helsinki, 00271 Finland

**Keywords:** Type 2 diabetes, Physical activity counselling, Primary Health Care, Glycated hemoglobin A, LDL cholesterol

## Abstract

**Background:**

Type 2 diabetes (T2D) has become a major public health threat; physical inactivity and obesity are both independent risk factors. Increasing daily physical activity (PA) significantly benefits treatment. Individual PA counselling is helpful for people with T2D, especially those with previous inactivity or with diabetes complications. This study evaluated factors contributing to effectiveness of PA counselling in primary health care (PHC) patients with T2D in a real-world setting and using data elicited from electronic health records (EHRs).

**Methods:**

All patients with T2D were offered the opportunity to participate in a PA program organized as part of basic PHC services in the Siun sote region in North Karelia, Finland, from October 2016 to December 2018. The study population consists of patients aged 19 to 87 years (n = 546). During the intervention information on possible other factors in addition to age and sex influencing the intervention effect such as amount of counselling sessions, changes in PA and patients´ motivation was gathered. Changes in the participants’ PA activity was generated by following the predefined rules from patient records and by assessing the descriptive documentation of activity patterns. The patients’ motivation level was assessed using a Likert scale.

**Results:**

Over 60% of participants who attended PA counselling more than three times increased their PA compared with 1% of participants with one counselling session. Of the whole intervention group, the participants experiencing the largest weight loss were those with an increased level of PA (-1.36 kg vs. -0.63 kg among those with no change in PA). Age, sex, and baseline motivation did not affect the change the PA nor the main intervention outcomes.

**Conclusions:**

Patients’ compliance with the intervention was reflected in the number of PA counselling sessions attended which in turn was seen as increased levels of PA as well as better treatment outcomes. In the implementation of lifestyle counselling interventions attention should be paid on sufficient amount and frequency of counselling sessions. The individually tailored PA counselling provided in PHC has similar effects regardless of sex and age.

## Background

People with type 2 diabetes (T2D) should engage regularly with moderate or vigorous intensity physical activity (PA) [1] as it improves the treatment outcomes of patients. T2D patients should switch from sitting to light exercise and interrupt long sitting sessions, because these measures have metabolic benefits [2, 3]. Consequently, lifestyle interventions, including PA, should play a key role in the treatment of chronic diseases like T2D to prevent complications as well as premature death [4–6].

There are many reasons including age, social and psychological factors, education, and a lack of knowledge for poor adherence in treatment and lifestyle changes in patient with T2D [7]. Adherence to treatments is usually lower in patients with chronic diseases compared with those with acute diseases. This is associated with the long-term nature of chronic diseases because the decline in adherence is most rapid after the first 6 months of therapy [8]. In addition, Leung et al. 2019 [9] have reported that adherence to PA interventions in the lifestyle modification program was lower than adherence to dietary interventions.

For lifestyle intervention programs to be successful, it is crucial that individuals adhere to the recommendations provided as well as possible. Patients who do not commit or drop out counselling prematurely get poorer outcomes [10]. Motivation, also described as willpower is often high in the initial stages of a lifestyle intervention program, however, maintaining the motivation is challenging [11]. Also, sex norms and expectations can influence engagement in PA as they can also be limiting factors for the possibilities to be physically active [12]. People with T2D are known to be less likely to engage in PA as a self-care activity, instead they more often commit to medication [13]. In addition, increased age leads to a decline in physical and social functioning and can affect self-care [14], for example commitment to lifestyle changes. Hence, a better understanding of the contributing factors of adherence for lifestyle counselling is vital to the successful counselling [10].

Several studies suggest that PA counselling can be successfully implemented in primary health care (PHC). It is cost-effective and practicable [15, 16]. PA counselling in PHC is recommended as a significant proportion of the population visits PHC. PHC is, therefore, an optimal environment for motivating patients to increase PA [17]. Currently, however, the opportunity for PA counselling is not sufficiently exploited in clinical work [16]. While healthcare professionals are generally positive about PA counselling, they cite a lack of time, knowledge, and skills, as well as doubts about the effectiveness of PA, as reasons for the poor utilization rate of PA counselling in clinical work [18]. PA counselling provided in health care systems (HCS) has been shown to increase PA slightly or moderately in different population groups. It is also important that leaders are engaged in supporting the provision of PA and other lifestyle counselling as part of services aiming at the prevention and treatment of chronic diseases [15].

There is evidence that prescribing exercise in PHC is a successful way of increasing PA during the 12-month follow-up period [16, 17]. Here, the focus should be on individual PA counselling that accommodates the current diagnosis, and counselling should be based on health status, PA history, risks, and individual preferences [4]. It is important that different approaches are used to promote PA and that interventions are tailored individually to each patient with T2D [19–22]. There is not yet sufficient evidence as to which methods and intervention components would be the most effective in increasing PA in PHC for people with T2D [16, 22, 23]. We studied the effectiveness of a PA counselling intervention delivered among patients with T2D in a ‘real world setting’ in PHC in North Karelia, Finland [24], and have evidenced some of the benefits of said intervention for treatment outcomes.

The aim of this study is to assess the factors contributing to the effectiveness of PA counselling in the intervention group. As the intervention was carried out in ‘real world’ settings as part of the service provision of the primary health care we were especially interested in how patients’ age, sex, attendance to the counselling sessions as well as their motivation affect the changes in PA and further in their treatment outcomes.

## Methods

### Study design and participants

All patients with T2D were offered the opportunity to participate in a PA program in the Siun sote region in North Karelia, Finland, from October 2016 to December 2018. Especially patients with very little physical activity were referred for PA counselling by nurses, physiotherapists, and physicians. Patients with T2D were also able to self-refer to the PA counselling program. The study population comprised patients aged 19 to 87 years with a diagnosis of T2D or prediabetes (n = 546). The follow- up time was from October 2016 to December 2019. While the follow-up period for measurements and laboratory results varied for each patient, based on when they were recruited, the follow-up period was at least one year for all patients. The practical arrangements of counselling and its content have previously been described in more detail [24]. Shortly counselling was given as part of PHC services by educators having training on physiotherapy and motivational interviewing. The intervention did not have any strictly standardized protocol but was tailored based on patients’ needs. Patients were given an opportunity to participate in various exercise experiments with the PA educator and co-operation was done with the PA services of municipalities.

The essential data on the patients’ circumstances and the content of the tailored PA counselling were recorded in the electronic patient information system. The entries were made using a premade template. The previously established International Classification of Diseases (ICD)-10 diagnoses and/or International Standard Classification of Primary Care (ICPC-2) codes are entered into the patient information system, and patients for this evaluation were identified using these codes and information on the type of the visit (appointment or other contact with PA educator).

This study was implementation research in primary care and carried out in ’real world’ setting. All measurement data were extracted from patient records and thus reflects normal processes in PHC. The study was carried out as a register-based study, which in accordance with the Finnish legislation (Act on secondary use of social and health data 26.4.2019/552), does not require ethics approval nor informed consent from the patients. However, the study has an ethics approval and a permission from the register keeper (Siun sote) to use register data in evaluation research.

### Measurements

As factors possibly influencing the intervention effect, we assessed patients` age and sex, the amount of attended counselling sessions, increase in PA and patients’ motivation. The number of counselling visits was identified from EHRs. Descriptive information about changes in the participants’ PA levels was produced from the material by assessing the written information describing the patients’ activity patterns, following predefined rules. Changes in PA levels were assessed by asking the patients during the counselling sessions (“How has your PA changed from previous habits?”) and categorized into the following categories for the purposes of analysis: 0 = unknown change in PA; 1 = no change in PA; and 2 = increased PA compared with the situation at the beginning of the intervention (see Table 1). The patients’ motivation level was assessed using a Likert scale. Patients were asked to evaluate their level of motivation for lifestyle change on a scale of 1 to 10, with these scores then categorized for the purpose of analysis into the following: 1–7 = low motivation; and 8–10 = high motivation. Motivation was assessed during the first PA counselling session.

Height and weight were recorded, and blood samples for Haemoglobin A1c (HbA1c) and low-density lipoprotein (LDL) were collected as part of the standard follow-up of T2D patients in PHC. The turbidimetric inhibition immune analysis method (TINIA) was used to analyze HbA1c. All samples were analyzed in the same regional laboratory Eastern Finland laboratory (ISLAB, https://www.islab.fi) which is an accredited laboratory and participates external quality surveys. Values were standardized to International Federation of Clinical Chemistry (IFCC) units.

### Statistical analyses

Basic statistics, such as frequency, percentage, mean, median, standard error (se), minimum and maximum, were used to describe the data. Changes during the intervention time in HbA1c, LDL, weight, and body mass index (BMI) were calculated by personal regression lines with time. Slopes were used as an estimate for changes in measurements per year. Differences in these yearly changes in measurements between sexes, age groups and motivation groups (low/high) were assessed by t-test. Analysis of variance was used to see if there is an association between yearly changes in measurements and change in PA or the amount of counselling sessions. Crosstabulation and chi-square test were used to assess the relationship between sex, age group and other contributing factors (PA, PA counselling, and motivation). IBM SPSS Statistics for Windows (Version 27.0) was used for the statistical analyses. P-values less than 0.05 were regarded as statistically significant [25].

## Results

The basic characteristics of the participants in the intervention group (n = 546) are shown in Table 1.

**Table 1:** Background characteristics of the participants

Variables	Measured % (n)	% (n)	Mean (sd)	Median (min,max)
Age (years)	100 (546)		57 (14)	59 (19,87)
Sex	100 (546)			
Female		61.4 (335)		
Male		38.6 (211)		
Weight, kg	78.6 (429)		102 (23)	99 (55,197)
BMI, kg/m^2^	78.2 (427)		36 (7.1)	35 (19,65)
HbA1c, mmol/ mol	69.2 (378)		52 (15)	48 (30,130)
LDL, mmol/l	79.1 (432)		2.8 (1.0)	2.6 (0.8,6.3)
PA, at the beginning of the intervention	82.6 (451)			
0 = no or very low		13.7 (62)		
1 = occasionally		27.6 (126)		
2 = regularly low intensity		40.6 (183)		
3 = regular moderate-intensity		14.6 (66)		
4 = very high activity		3.1 (14)		
Motivation, at the beginning of the intervention	36.6 (200)			
0 = unknown		63.4 (346)		
1 = low motivation		3.1 (17)		
2 = high motivation		33.5 (183)		

Table 2 presents the changes in weight, BMI, HbA1c, and LDL levels by sex and age group. The data on changes in HbA1c values was available for approximately 69% of patients (n = 378) in the intervention group, LDL values for approx. 69% of patients (n = 376), BMI values for 78% of patients (n = 427) and change in weight values for approximately 79% of patients (n = 429). Although a decline in outcome measures was observed in all groups, no significant differences were observed between men and women, or the age groups analyzed.

**Table 2:** Changes in HbA1c, LDL, BMI levels, and weight by sex and age group

	Sex	Age groups, years	% (n)	Mean	Std. Error mean	P-value for the difference
Change in	F		58 (221)	-0.75	0.52	0.497
HbA1c, mmol/mol	M		42 (157)	-1.35	0.752	
		< 65	56 (213)	-1.02	0.691	0.940
		≥ 65	44 (165)	-0.96	0.449	
Change in	F		60 (225)	-0.2	0.063	0.495
LDL, mmol/l	M		40 (151)	-0.14	0.035	
		< 65	58 (218)	-0.21	0.064	0.384
		≥ 65	42 (158)	-0.14	0.037	
Change in	F		61 (261)	-0.43	0.152	0.313
BMI, kg/m^2^	M		39 (166)	-0.67	0.172	
		< 65	64 (275)	-0.61	0.165	0.315
		≥ 65	36 (152)	-0.37	0.122	
Change in	F		61 (263)	-1.28	0.408	0.435
Weight, kg	M		39 (166)	-1.78	0.471	
		< 65	64 (276)	-1.71	0.446	0.310
		≥ 65	36 (153)	-1.05	0.322	

Hemoglobin A1c (HbA1c), low-density lipoprotein (LDL), body mass index (BMI). Statistical methods: T-test

In the intervention group, 18% of participants (n = 98) increased PA and 39% participants (n = 215) did not have a noticeable change in PA. There was no significant difference in the change of PA between the sex and analyzed age groups. The change in weight was greater in those who increased PA and in those whose PA was unknown compared to those having no change in PA. The difference between the groups was statistically significant (p = 0.046) (Table 3).

**Table 3:** Change in PA by sex and age and associations with change in treatment outcome indicators

Change in PA level	PA change unknown	No change in PA	Increased PA	Total	P-value for the difference between the groups
Total, %(n)	43 (233)	39 (215)	18 (98)	100 (546)	
Female, %(n)	43 (146)	38 (126)	19 (63)	100 (335)	0.546^a^
Male, %(n)	41 (87)	42 (89)	17 (35)	100 (211)	
Age groups,					
years					
< 65, %(n)	43 (152)	37 (131)	20 (68)	100 (351)	0.323 ^a^
≥ 65, %(n)	42 (81)	43 (84)	15 (30)	100 (195)	
Change in HbA1c, mmol/mol					
mean (se)	-1.77 (0.81)	-0.67 (0.61)	0.16 (0.56)	-1.00 (378)	0.251^b^
Change in LDL, mmol/l					
mean (se)	-0.19 (0.05)	-0.18 (0.08)	-0.15 (0.04)	-0.18 (376)	0.932 ^b^
Change in BMI, kg/m^2^					
Mean (se)	-0.79 (0.18)	-0.28 (0.18)	-0.43 (0.24)	-0.52 (427)	0.121 ^b^
Change in weight, kg					
mean (se)	-2.33 (0.50)	-0.63 (0.44)	-1.36 (0.78)	-1.47 (429)	0.046 ^b^

Hemoglobin A1c (HbA1c), low-density lipoprotein (LDL), body mass index (BMI). Statistical methods: ^a^ Chi-square test, ^b^ Analysis of variance and t-test otherwise

Table 4 indicates the number of PA counselling sessions by age group and sex and the association of the number of PA counselling on changes in PA, HbA1c and LDL levels, BMI, and weight. Data on PA counselling sessions was identified for 521 intervention group participants. Of these, 41% (n = 215) attended PA counselling on average 2–3 times. Men and women and younger and older patients attended similarly to the counselling sessions. The majority of those with increased PA had attended PA counselling more than three times. The associations between the number of attended sessions and anthropometric and biological treatment outcome variables did not come statistically significant.

**Table 4:** The association of PA counselling attendance on PA, HbA1c, LDL levels, BMI, and weight

No. of PA counselling sessions	No information about sessions	1 session	2–3 sessions	More than 3 sessions	P-value for the difference between the groups
Female, %(n)	4 (14)	29 (98)	39 (130)	28 (93)	0.787^a^
Male, %(n)	5 (11)	26 (54)	40 (85)	29 (61)	
Age groups, years					
< 65, % (n)	4 (15)	29 (101)	38 (133)	29 (102)	0.726^a^
≥ 65, % (n)	5 (10)	26 (51)	42 (82)	27 (52)	
PA change unknown, %(n)	11 (25)	57 (133)	24 (57)	8 (18)	
No change in PA, %(n)	0 (0)	8 (18)	57 (122)	35 (75)	0.001^a^
Increased PA, %(n)	0 (0)	1 (1)	37 (36)	62 (61)	
Change in					
HbA1c, mmol/mol					
mean (se)		-2.18 (1.03)	-0.51 (0.56)	-0.54 (0.74)	0.223^b^
Change in LDL, mmol/l					
mean (se)		-0.19 (0.06)	-0.22 (0.08)	-0.11 (0.03)	0.469^b^
Change in BMI, kg/m^2^					
mean (se)		-0.43 (0.18)	-0.48 (0.22)	-0.52 (0.18)	0.961^b^
Change in Weight, kg					
mean (se)		-1.33 (0.48)	-1.22 (0.55)	-1.53 (0.55)	0.915^b^

Hemoglobin A1c (HbA1c), low-density lipoprotein (LDL), body mass index (BMI). Statistical methods: ^a^ Chi-square test and ^b^Analysis of variance

Table 5 shows the association of motivation on changes in PA, HbA1c, LDL levels, BMI, and weight. In the intervention group, motivation estimates were found for 37% of participants (n = 200). Of these, 67% (n = 134) and 33% (n = 66) had estimated their motivation to be high and low at the beginning of the intervention, respectively. Participants who had estimated their motivation for lifestyle change to be high had demonstrated a somewhat greater change in laboratory measurements, BMI, and weight than the participants who had assessed their motivation to be lower. However, the differences were not statistically significant.

**Table 5:** Association of motivation on changes in PA, HbA1c, LDL, BMI levels and weight

	Low motivation	High motivation	Total (n)	P-value for the difference between the groups
PA change unknown, %(n) No change in PA, %(n) Increased PA, %(n)	27 (18) 55 (36) 18 (12)	37 (50) 42 (56) 21 (28)	200	0.219^a^
Change in HbA1c, mmol/mol mean (se)	-0.99 (1.04)	-1.67 (0.96)	151	0.672^b^
Change in LDL, mmol/l, mean (se)	-0.08 (0.06)	-0.14 (0.47)	150	0.445^b^
Change in BMI, kg/m^2^ mean (se)	-0.09 (0.37)	-0.65 (0.24)	157	0.202^b^
Change in Weight, kg mean (se)	-0,17 (1.05)	-1.77 (0.58)	158	0.159^b^

Low motivation 1–7 and high motivation 8–10. Hemoglobin A1c (HbA1c), low-density lipoprotein (LDL), body mass index (BMI). Statistical methods: ^a^ Chi-square test and ^b^ T-test

## Discussion

This study assessed the factors influencing the effectiveness of PA counselling provided in a ‘real world’ setting in primary care patients with T2D. The primary finding of this study is that the PA level of participants who attended PA counselling more than three times increased the most. The results of the effectiveness evaluation have been previously reported [24] and demonstrate that PA counselling in PHC, even with modest increase in exercise, offers significant benefits in the treatment of T2D. The reduction in weight was greater in intervention group participants who had increased PA compared with participants who had not changed their PA habits. No significant associations between the amount of counselling or recorded changes in PA were observed with biological outcome measures. As the overall effect of the intervention has been shown to be positive to those outcome factors [24] the current finding can be due to reduced power in the analyses when further data stratifications and additions of explanatory variables were made, which also easily increase the amount of missing data.

In the present study, intervention group participants who attended PA counselling more than three times had the highest increase in PA levels. Moreover, the change in weight was greater in participants who were more physically active. The research on effects of different doses of PA interventions among patients with T2D is scarce. However, the study by Bauman et al. [26] showed that high dose of behavioral treatment including 24 sessions over 6 months resulted in significantly greater reductions in HbA1c and fasting blood glucose than the low-dose and control conditions among patients with obesity or prediabetes. In our study, positive effects were observed with much less attended sessions. Ngandu et al. [27] reported from the multifactorial lifestyle modification intervention among patients at high risk of memory disorders that active participation was associated with better outcomes in cognitive decline. It is essential that lifestyle interventions are intensive and there is sufficient support for adherence. However, there is still very little research on adherence to lifestyle modification and more detailed studies are needed to increase knowledge about the factors related to successful lifestyle modification [28].

Our results also showed that the intervention results did not differ between sex or age groups. Hays et al. [29] and Nelson et al. [30] have shown that PA is positively associated with younger age and male sex in patients with T2D. Additionally, Kelly et al. [31] revealed sex differences in the PA levels of older adults with T2D in their study, in which they assessed the number of daily steps taken by older adults with T2D. Conversely, however, in their systematic review, Haywood and Sumithran [32] stated that the lifestyle interventions had similar weight loss effects in both older and younger people.

Many studies have reported that the cornerstones of prevention and treatment of T2D are a healthy diet, PA, and weight loss before starting medication [33–36]. It has been shown that intensive lifestyle interventions even though not exactly reaching the equivalent glycemic control compared with standard care can result in considerably good glucose control [33]. Although medication is effective in improving HbA1c levels in patients with T2D, it can result in side effects, increased financial costs, and impaired quality of life [33]. Consequently, in addition to medication, measures that support healthier lifestyles should always be key components of care. However, a significant proportion of adults do not achieve current PA recommendations [37]. In fact, adults with T2D are less likely to engage in regular PA than the general adult population [38]. Therefore, it is important to invest in lifestyles that can promote and maintain glycemic control almost to the same extent as medication [33].

Individual PA counselling is helpful for people with T2D, especially for those with previous physical inactivity or with diabetes complications [39]. Kirk et al. [40] have reported that PA counselling effectively promotes PA during a 12-month follow-up period in people with T2D. According to them, PA counselling was more effective than a standard exercise leaflet in promoting and maintaining PA in people with T2D [40] also addressing the importance of intensity of the intervention.

As the population age and the incidence of both prediabetes and diabetes increases, it is important to implement and evaluate cost-effective methods for prevention and treatment that are adjusted for HCS [16, 41, 42]. Studies by Rossen et al. [4] and Myers et al. [43] have shown that a small improvement in the physical fitness of a person with T2D reduces the risk of overall mortality. Stevens et al. [44] and Lee et al. [45] have shown that even modest increases in PA offer significant benefits in the treatment of T2D. PA programs should be an integral part of the treatment of T2D [38, 40]. Matthews et al. [20] have stated that PA interventions for adults with T2D can be effectively translated into an everyday setting. They have shown that effective interventions can be delivered by a variety of trained staff/peers in a variety of settings [20]. However, PA counselling conducted in primary care settings has often been found to be infrequent and inadequate [46].

In this study, motivation was assessed during the first PA counselling session. In the intervention group, 67% of participants had estimated their motivation for lifestyle change to be high, but no statistically significant association was found between motivation levels and changes in laboratory measurements, BMI, or weight, despite the direction of change indicating this. Information on motivation could only be collected from some of the participants and the number of observations might not be sufficient to show the possible differences. Motivation levels were also not found to be associated with changes in PA. Lack of motivation [47] and lack social support [48] for exercise are often barriers to increasing PA patient with T2D. Thus, understanding T2D patients’ motivation for behavior change is considered central to successful patient-centered care [49]. However, motivating patients for good self-management of diabetes is one of the greatest challenges in health care [50]. People are often interested in their own health, but health care professional cannot help a person to make lifestyle changes unless he/she is motivated to do so. Thus, health care professionals should focus on maintaining the patients’ motivation. Counselling should encourage the patient to moderate lifestyle changes and address individual tailoring guidance [51].

Diabetes treatment guidelines recommend theory-based, patient-centered care and advocate the provision of support for patient motivation [52]. Research amongst people with T2D has shown that autonomous motivation is positively associated with healthy lifestyles such as PA [53], dietary self-care [54, 55], and continuous improvement of physical health, such as through diabetes control [56]. Health care settings can promote the internalization process by supporting patients’ autonomous motivation and self-care competence [56]. However, motivation for lifestyle change immediately following T2D diagnosis can be relatively low [52]. Sebire et al. [52] stated in their study that some participants were reluctant to change and articulated a passivity towards any change. In addition, ignoring one’s diabetes, feeling helpless, and not able to change one’s current way of life, as well as a disbelief in the recommended therapies bringing about health benefits were among other reasons for low motivation. Changes in behavior may be more sustainable if patients with T2D can be supported to internalize their motivation to the point of identifying a personal benefit or integrating change as a part of a meaningful way of life [50].

In the study by Koponen et al. [57] the three central variables of self-determination theory were studied. They observed that perceived autonomy support, autonomous motivation and self-care competence were associated with successful weight management among patients with T2D in PHC. They addressed the role of physicians and other health care professionals in motivating the patients and in understanding the value of the health benefits in weight management. They concluded that interventions based on self-determination theory together with autonomy supportive care environments can strengthen patients’ autonomous motivation and self-care competence. In our study, information on the level of motivation was only available at the beginning of the intervention, i.e., we do not know how motivation changed during the intervention and what effect it has had on patient outcomes.

### Strengths and limitations

This study was carried out in a ‘real world setting’ and, thus, the results are directly generalizable with the normal processes in health care. In the absence of a traditional study design in this study, the study participation did not influence patients’ behavior. All measurement data were extracted from patient records. The absence of separate data recording resulted in the measurement data remaining partly incomplete. The data on PA sessions and laboratory measures represent the actual activities in health care, which do not occur according to a standard, strict protocol. The data that are dependent on the coverage of assessments and recording by professionals, such as information on the level of PA or motivation, remain even more scarce, which has an influence on the power of the analyses.

## Conclusions

This study shows that PA counselling in PHC is relevant in the treatment of T2D. Increasing PA is likely to bring health benefits and improve quality of life for patients with T2D. The results indicate that individually tailored PA counselling provided in PHC has similar effects regardless of sex and age. Only the number of counselling sessions and the achieved increase in PA significantly affected the outcomes. In addition, this study slightly indicated that patients with T2D who are more motivated to make lifestyle changes achieve better results. The observed changes in both PA and biological measures, especially weight, were modest. However, as shown in many studies PA is many ways beneficial for the treatment of T2D patients without big improvements in single outcome measures. This study also showed how challenging it is to achieve changes in lifestyles. Continuity and frequency of counselling matters emphasizing the importance of resource availability and any measures intended to increase the adherence of patients.

## Data Availability

The health records data analyzed in the current study are confidential and, in accordance with the Personal Data Act in Finland, cannot be made publicly available in order to protect the privacy of the patients. According to the legislation, register authorities give permissions to use register data including sensitive individual information (e.g., health data) to study specified research questions to named individuals who have signed a pledge of secrecy and they are not permitted to forward it to other researchers. In issues related to the data availability the corresponding author Tuula Martiskainen can be contacted.

## Abbreviations

ACSM: American College of Sports Medicine
ADA: American Diabetes Association
BMI: Body mass index
CV: Cardiovascular
CVD: Cardiovascular disease
DSE: Diabetes support and education
EHRs: Electronic health records
ET: Exercise training
HbA1c: Haemoglobin A1c
HCS: Health care systems
ICD-10: International Classification of Disease diagnoses
ICPC-2: International Standard Classification of Primary Care codes
ILI: Lifestyle intervention
LDL: Low-density lipoprotein
LTPA: Leisure-time physical activity
MI: Motivational interview
PA: Physical activity
PAD: Peripheral arterial disease
PHC: Primary health care
SDT: Self-determination theory
SWM: Success in weight management
T2D: Type 2 Diabetes
TINIA: Turbidimetric inhibition immune analysis
